# Analysis of mitochondrial genomes resolves the phylogenetic position of Chinese freshwater mussels (Bivalvia, Unionidae)

**DOI:** 10.3897/zookeys.812.29908

**Published:** 2019-01-03

**Authors:** Rui-Wen Wu, Xiong-Jun Liu, Sa Wang, Kevin J. Roe, Shan Ouyang, Xiao-Ping Wu

**Affiliations:** 1 School of Life Sciences, Nanchang University, Honggutan-New-District, Nanchang 330031, China Nanchang University Nanchang China; 2 School of Resource, Environment and Chemical Engineering, Nanchang University, Nanchang 330031, China Iowa State University Ames United States of America; 3 Poyang Lake Key Laboratory of Environment and Resource Utilization (Nanchang University), Ministry of Education, Nanchang 330031, China Nanchang University Nanchang China; 4 Department of Natural Resource Ecology and Management, Iowa State University, Ames, 50011, United States of America Iowa State University Ames United States of America

**Keywords:** China, classification, freshwater, F-type mitogenome, mussel

## Abstract

The Yangtze River basin is one of the most species-rich regions for freshwater mussels on Earth, but is gravely threatened by anthropogenic activities. However, conservation planning and management of mussel species has been hindered by a number of taxonomic uncertainties. In order to clarify the taxonomic status and phylogenetic position of these species, mitochondrial genomes of four species (*Acuticostachinensis*, *Schistodesmuslampreyanus*, *Cuneopsisheudei* and *Cuneopsiscapitatus*) were generated and analyzed along with data from 43 other mitogenomes. The complete F-type mitogenomes of *A.chinensis*, *S.lampreyanus*, *C.heudei*, and *C.capitatus* are 15652 bp, 15855 bp, 15892 bp, and 15844 bp, respectively, and all four F-type mitogenomes have the same pattern of gene arrangement. ML and BI trees based on the mitogenome dataset are completely congruent, and indicate that the included Unionidae belong to three subfamilies with high bootstrap and posterior probabilities, i.e., Unioninae (*Aculamprotula*, *Cuneopsis*, *Nodularia*, and *Schistodesmus*), Anodontinae (*Cristaria*, *Arconaia*, *Acuticosta*, *Lanceolaria*, *Anemina*, and *Sinoanodonta*), and Gonideinae (*Ptychorhynchus*, *Solenaia*, *Lamprotula*, and *Sinohyriopsis*). Results also indicate that *A.chinensis* has affinities with *Arconaialanceolata* and *Lanceolariagrayii* and is a member of the subfamily Anodontinae.

## Introduction

The freshwater mussel family Unionidae is the most species-rich family within the order Unionida, including more than 620 species representing 142 genera (Graf and Cummings 2007; Bogan 2008). The Unionidae is widely distributed, and its members are found on all continents, with the exception for Antarctica (Graf and Cummings 2007; Bogan 2008; Lopes-Lima et al. 2017a). Unfortunately, freshwater mussels are one of the most threatened animal groups in the world, due to habitat destruction, commercial exploitation, and water pollution (Lydeard et al. 2004; Vaughn et al. 2010; Lopes-Lima et al. 2014; Wu et al. 2017a).

Well-supported phylogenetic hypotheses for the Unionidae are crucial for understanding the evolutionary history and biogeography of its genera (e.g., Roe 2013; Graf et al. 2015), for formulating reliable classifications (e.g., Campbell et al. 2005), and for developing conservation priorities (Lopes-Lima et al. 2017b, 2018). Advances in developing improved phylogenetic hypotheses for the Unionidae have occurred in the past several decades (Davis 1984; Lydeard et al. 1996; Nagel and Badino 2001; Hoeh et al. 2001, 2002; Giribet and Wheeler 2002; Graf 2002; Campbell et al. 2005; Zanatta and Murphy 2006; Graf and Cummings 2007; Campbell and Lydeard 2012a, b; Froufe et al. 2014; Prié and Puillandre 2014; Graf et al. 2015; Pfeiffer and Graf 2015). Most of these studies have focused on North American, Australian, and European taxa, although more recently, African (Whelan et al. 2011; Graf 2013; Elderkin et al. 2016) and Asian (Huang et al. 2002; Zhou et al. 2007; Huang et al. 2013; Bolotov et al. 2017a, b) taxa have been included, and a global phylogenetic framework of the Unionidae has recently been established (Bolotov et al. 2017a; Lopes-Lima et al. 2017a). Despite these advances, the incorporation of Asian taxa into unionid phylogenetic hypotheses, particularly those from China has lagged.

The middle and lower reaches of the Yangtze River are a diversity hotspot for unionids in East Asia (Graf and Cummings 2007; He and Zhuang 2013; Zieritz et al. 2017), and this region may harbor as many as 15 unionid genera (Wu et al. 2000; Shu et al. 2009; Wu et al. 2017a). As with North American freshwater mussels, much of the early descriptive work on Chinese taxa occurred during the latter part of the 19^th^ Century (Heude 1875, 1877a, b, 1878, 1879, 1880a, b, 1881, 1883, 1885). Pierre Marie Heude was a Jesuit priest who collected freshwater and terrestrial mollusks in China. During a ten-year period between 1882 and 1902, Heude described close to 600 species including 140 freshwater mussel species (Johnson 1973). However, the validity and classification of many of these species were called in to question by Simpson (1900, 1914) and Haas (1969). Simpson (1900, 1914) presented a modified classification based on anatomical information such as marsupium size and shape, larval type and umbo sculpture in addition to conchological characters. Simpson condensed the number of Chinese freshwater mussels down to 85 species in 14 genera and placed them into two subfamilies, the Unioninae and the Hyriinae. Haas (1969) further revised the classification of the Unionidae and reduced the number of Chinese unionids to 56 species and subspecies in 20 genera, and placed them into four subfamilies: Unioninae, Quadrulinae, Anodontinae and Lampsilinae. After 1949, Chinese malacologists (e.g., Lin 1962; Tchang et al. 1965a, b; Liu et al. 1964, 1979, 1980, 1982; Wu et al. 2000) conducted a substantial amount of work on the classification of the Unionidae, and placed Chinese species into either the Unioninae or Anodontinae, based on the presence or absence of hinge teeth. In the 1990s, malacologists began to refocus their attention on the soft anatomy and changes to the classification, based on the shape of the glochidia and type of marsupium were made (Wei and Fu 1994; Wu et al.1999a, b; Shu et al. 2012). Despite these advances, the higher-level taxonomy of Chinese unionids was not updated, and only the subfamilies Unioninae and Anodontinae remained in the revised system.

At the beginning of this century, Chinese researchers investigated the molecular systematics of the Unionidae and made great progress revising the earlier classifications (Huang et al. 2002; Wang et al. 2013; Ouyang et al. 2011, 2015; Huang et al. 2013, 2015, 2018; Song et al. 2016; Zhou et al. 2007, 2016a, b; Wu et al. 2016, 2017b). However, there continued to be many discrepancies regarding the classification of genera (Table 1). Most recently, Lopes-Lima et al. (2017a) constructed a phylogenetic framework for the worldwide Unionidae; however, it only contained 17 Chinese freshwater mussel species. Wu et al. (2018b) generated a phylogeny based on portions of the mitochondrial COI and ND1genes that included 34 Chinese unionids. While the resultant trees from these studies resolved a number of relationships, branch support values at certain nodes were low, and the placements of some genera (*Sinohyriopsis* and *Lepidodesma*) were not clarified.

**Table 1.** Chinese freshwater mussels (Unionidae) systematic taxonomy history. Shaded genera indicate classification disputes.

Genus	Liu et al. 1979	Huang et al. 2002	Zhou et al. 2007	Ouyang et al. 2011	Huang et al. 2013	Ouyang et al. 2015	Wu et al. 2018b	This study
*Aculamprotula* Wu et al., 1999	–	–	Unioninae	Unioninae	Unioninae	Unioninae	Unioninae	Unioninae
*Sinanodonta* Modell, 1944	Anodontinae	Anodontinae	Anodontinae	Anodontinae	–	Anodontinae	Anodontinae	Anodontinae
*Cristaria* Schumacher, 1817	Anodontinae	Anodontinae	Anodontinae	–	Anodontinae		Anodontinae	Anodontinae
*Cuneopsis* Simpson, 1900	Unioninae	Unioninae	Unioninae	Unioninae	–	Unioninae	Unioninae	Unioninae
*Schistodesmus* Simpson, 1900	Unioninae	Unioninae	Unioninae	Unioninae	–	Unioninae	Unioninae	Unioninae
*Nodularia* Conrad, 1853	Unioninae	Unioninae	Unioninae	Unioninae	–	Unioninae	Unioninae	Unioninae
*Anemina* Haas, 1969	Anodontinae	Anodontinae	–	Anodontinae	–	Anodontinae	Anodontinae	Anodontinae
*Acuticosta* Simpson, 1900	Unioninae	Unioninae	Unioninae	Unioninae	–	Unioninae	Anodontinae	Anodontinae
*Arconaia* Conrad, 1865	Unioninae	Unioninae	Unioninae	–	–	–	Anodontinae	Anodontinae
*Lamprotula* Simpson, 1900	Unioninae	Ambleminae	Ambleminae	Ambleminae	–	Ambleminae	Gonideinae	Gonideinae
*Lanceolaria* Conrad, 1853	Unioninae	Unioninae	Unioninae	Unioninae	–	Unioninae	Anodontinae	Anodontinae
*Lepidodesma* Simpson, 1896	Anodontinae	Unioninae	–	–	–	–	**Incertae sedis**	**Incertae sedis**
*Ptychorhynchus* Simpson, 1900	–	Ambleminae	–	–	–	–	Gonideinae	Gonideinae
*Solenaia* Conrad, 1869	Anodontinae	Ambleminae	–	Ambleminae	Gonideinae	Ambleminae	Gonideinae	Gonideinae
*Sinohyriopsis* Starobogatov, 1970	Unioninae	Ambleminae	Ambleminae	Ambleminae	–	Ambleminae	**Incertae sedis**	Gonideinae

The purpose of this study was to clarify the taxonomic status and phylogenetic position of Chinese Unionidae using the DNA sequences of mitochondrial genomes to infer phylogenetic relationships. Phylogenetic hypotheses based on the analysis of mitochondrial genomes of unionids are becoming more common (Walker et al. 2006; Huang et al. 2013, 2018; Burzyński et al. 2017). In the Unionoida, Mytiloida, and Veneroida, an unusual mode of mitochondrial DNA transmission termed Doubly Uniparental Inheritance (DUI) occurs, in which two distinct, tissue-specific and gender-associated mitogenomes (i.e., F-type and M-type) (Breton et al. 2007) are present. For the remainder of this paper, all references to mitogenomes refer to the F-type mitogenome.

In this study, we sequenced and described the complete mitogenomes of four Chinese unionids: *Acuticostachinensis* (Lea, 1868), *Schistodesmuslampreyanus* (Baird & Adams, 1867), *Cuneopsisheudei* (Heude, 1874), and *Cuneopsiscapitatus* (Heude, 1874), with the aim of combining these new genome sequences with existing mitochondrial genomes to develop a phylogenetic framework for the Chinese Unionidae. In addition, we were particularly interested in determining the taxonomic position of the genus *Acuticosta*. This genus was erected by Simpson (1900) and *Acuticostachinensis* (Lea, 1868) was used as the type species. The genus *Acuticosta* has been placed in a number of unionid subfamilies including the Hyriinae (Simpson, 1900), Unioninae (Liu 1979), Acuticostinae (Prozorova et al. 2005), and Unioninae (Huang et al. 2002, Graf and Cummings 2007, Zhou et al. 2007, Ouyang et al. 2011, 2015), and most recently, the Anodontinae (Wu et al. 2018b).

## Materials and methods

### Taxon sampling, mitochondrial genome sequencing, and assembly

**Figure 1.**
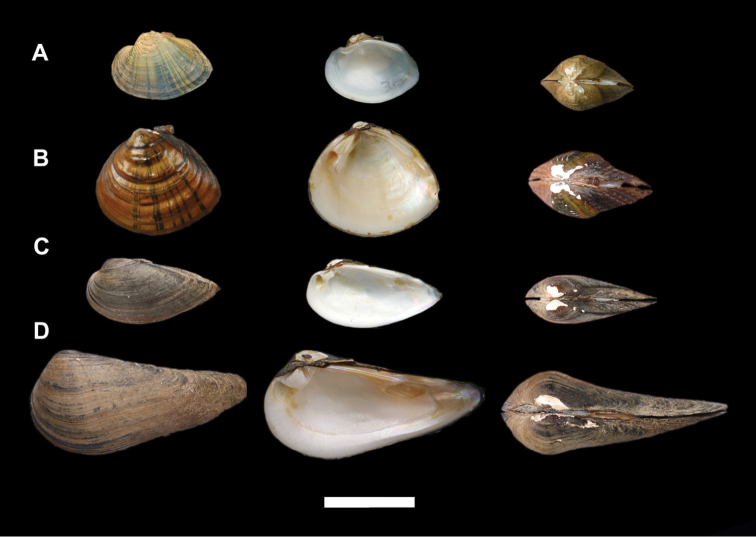
Shells of the unionids species in this study. **A***Acuticostachinensis* (Lea, 1868) **B***Schistodesmuslampreyanus* (Baird & Adams, 1867) **C***Cuneopsisheudei* (Heude, 1874) **D***Cuneopsiscapitatus* (Heude, 1874). Scale bar: 4 cm. Photogaphs R-W Wu.

Samples of four species were collected from Poyang Lake (28°47.84'N; 116°2.03'E) in Jiangxi Province, China (Figure 1), and specimens were preserved and vouchers deposited in the Biological Museum of Nanchang University. Information for primers used for PCR amplification of F-type mitogenomes can be found in Table 2. Complete mitogenomes were sequenced and annotated according to our previous study (Wu et al. 2016).

**Table 2.** Primers used for PCR amplification of female *Acuticostachinensis*, *Schistodesmuslampreyanus*, *Cuneopsisheudei*, and *Cuneopsiscapitatus* mitochondrial genomes.

Fragment	Primer name	Primer sequence (5’ to 3’)	Length
COI (universal primer)	LCO1490	GGTCAACAAATCATAAAGATATTGG	~700 bp
	HCO2198	TAAACTTCAGGGTGACCAAAAAATCA	
16S (universal primer)	16SarL	CGCCTGTTTATCAAAAACAT	~500 bp
	16SbrH	CCGGTCTGAACTCAGATCACGT	
ND1 (universal primer)	Leu-uurF	TGGCAGAAAAGTGCATCAGATTAAAGC	~1000 bp
	LoGlyR	CCTGCTTGGAAGGCAAGTGTACT	
COI→ND1 (*A.chinensis*)	ZGCNH	TTGGGACTGGCTGGAC	~500 bp
	ZGCNR	TTACTAGGAGCTATTCGAGC	
	2ZGCNH	GAGTCTTGGGGTTTATTGT	~1400 bp
	2ZGCNR	AGTAGAAAGACCAAAACCG	
	3ZGCNH	CAGTTCGGTGTTATCTTCAT	~3400 bp
	3ZGCNR	TGGCTAGTAGTGATTCTTGC	
ND1→16S (*A.chinensis*)	ZGN1H	CGAAGCCTGACAATGTCTA	~4500bp
	ZGN1R	TATCGAAAGTTGGGTTTGC	
16S→COI (*A.chinensis*)	ZG1CH	CTAGTGTTGCCTTTCACTG	~5200 bp
	ZG1CR	AGACAAGGGAGGATAAACC	
COI→ND1 (*S.lampreyanus*)	SXCNH	CTGGTTGGACGGTGTATC	~3200 bp
	SXCNR	ATAGCCATCCCAGTAGCC	
	2SXCNH	GTTATACTCTTCCGATCATCCT	~2100 bp
	2SXCNR	AACCAGCACAGAACTCAATA	
ND1→16S (*S.lampreyanus*)	SXN1H	GAGATGGTTTGAGCTATGG	~4500 bp
	SXN1R	CGATGTTGGCTTAAGGATA	
16S→COI (*S.lampreyanus*)	SX1CH	TTCCTAGTCTTGCCATTCA	~3600 bp
	SX1CR	GCAGGCACAAGTAATCAAA	
COI→ND1 (*C.heudei*)	YTCNH	TCTGGTGATGCCAATAATGA	~6200 bp
	YTCNR	TCCCCTCCTTTATAGTTTCA	
ND1→16S (*C.heudei*)	YTN1H	TGTCTCTGCGAGGATTACT	~1300 bp
	YTN1R	ACATAAGTGCAACCGCTAT	
	2YTN1H	TTCTGCCACCTTGCTTCA	~3300 bp
	2YTN1R	GGCTGACTCATACGAACCAT	
16S→COI (*C.heudei*)	YT1CH	TTACTGGTTCCAAGATTGC	~5600 bp
	YT1CR	AATCAAACCAGGAGATCGT	
COI→ND1 (*C.capitatus*)	JSCNH	GTTGCTGAGCGTATTCCTT	~5300 bp
	JSCNR	CTTTGACTTTGCAGAGGGA	
ND1→16S (*C.capitatus*)	JSN1H	GTATTTGGAGTTGGATGATC	~4700 bp
	JSN1R	GAATGGCAAGACTAGGAATA	
16S→COI (*C.capitatus*)	JS1CH	TATTCCTAGTCTTGCCATTC	~5000 bp
	JS1CR	CAATAATCTTCCAGGTTGAC	

### Dataset construction

We downloaded all published unionid mitogenomes from GenBank (as of March 2018), and combined them with the four mitogenomes generated in this study for a total of 41 unionid mitogenomes (22 Chinese taxa). In addition, we included additional genomes, also downloaded from GenBank, from the Margaritiferidae (four species), Iridinidae (one species), and Hyriidae (one species) as out-groups for the phylogenetic analysis (Table 3).

**Table 3.** F-type mitochondrial genomes used in this study.

Taxon	GenBank accession number	Reference
UNIONIDAE		
Ambleminae		
*Quadrulaquadrula* (Rafinesque, 1820)	FJ809750	Breton et al. 2009
*Venustaconchaellipsiformis* (Conrad, 1836)	FJ809753	Breton et al. 2009
*Potamilusalatus* (Say, 1817)	KU559011	Wen et al. 2017
*Leptodealeptodon* (Rafinesque, 1820)	NC_028522	Feng et al. 2016
*Toxolasmaparvum* (Barnes, 1823)	HM856639	Breton et al. 2011
*Lampsilisornata* (Conrad, 1835)	NC_005335	Serb and Lydeard 2003
Gonideinae		
*Pronodulariajapanensis* (Lea, 1859)	AB055625	Unpublished
*Lamprotulaleaii* (Griffith & Pidgeon, 1833)	NC_023346	Chen et al. 2012
*Ptychorhynchuspfisteri* (Heude, 1874)	KY067440	Zhou et al. 2016a
*Potomidalittoralis* (Cuvier, 1798)	NC_030073	Froufe et al. 2016
*Solenaiaoleivora* (Heude, 1877)	NC_022701	Huang et al. 2015
*Solenaiacarinatus* (Heude, 1877)	NC_023250	Huang et al. 2013
*Sinohyriopsisschlegelii* (Martens, 1861)	HQ641406	Unpublished
*Sinohyriopsiscumingii* (Lea, 1852)	NC_011763	Unpublished
Anodontinae		
*Acuticostachinensis* (Lea, 1868)	MH919390	This study
*Arconaialanceolat*a (Lea, 1856)	KJ144818	Wang et al. 2014
*Lanceolariagrayana* (Lea, 1834)	NC_026686	Unpublished
*Pyganodongrandis* (Say, 1829)	FJ809754	Breton et al. 2009
*Utterbackiapeninsularis* Bogan & Hoeh, 1995	HM856636	Breton et al. 2011
*Utterbackiaimbecillis* (Say, 1829)	HM856637	Breton et al. 2011
*Lasmigonacompressa* (Lea, 1829)	NC_015481	Breton et al. 2011
*Anodontaanatina* (Linnaeus, 1758)	NC_022803	Soroka et al. 2015
*Sinanodontawoodiana* (Lea, 1834)	HQ283346	Soroka et al. 2010
*Sinanodontalucida* (Heude, 1877)	KF667529	Song et al. 2016
*Aneminaarcaeformis* (Heude, 1877)	KF667530	An et al. 2016
*Aneminaeuscaphys* (Heude, 1879)	NC_026792	Xue et al.2016
*Cristariaplicata* (Leach, 1814)	KM233451	Wang et al. 2016
Unioninae		
*Lepidodesmalanguilati* (Heude, 1874)*	NC_029491	Zhou et al. 2016b
*Schistodesmuslampreyanus* (Baird & Adams, 1867)	MH919388	This study
*Cuneopsispisciculus* (Heude, 1874)	NC_026306	Han et al. 2016
*Cuneopsisheudei* (Heude, 1874)	MH919389	This study
*Cuneopsiscapitatus* (Heude, 1874)	MH919387	This study
*Nodulariadouglasiae* (Griffith & Pidgeon, 1833)	NC_026111	Unpublished
*Uniodelphinus* Spengler, 1793	KT326917	Fonseca et al. 2017
*Uniopictorum* (Linnaeus, 1758)	NC_015310	Soroka et al. 2010
*Uniocrassus* Retzius, 1788	KY290446	Burzyński et al. 2017
*Uniotumidus* Retzius, 1788	KY021076	Soroka et al. 2018
*Aculamprotulatortuosa* (Lea, 1865)	NC_021404	Wang et al. 2013
*Aculamprotulascripta* (Heude, 1875)	MF991456	Wu et al. 2017b
*Aculamprotulacoreana* (Martens, 1886)	NC_026035	Lee et al. 2016
*Aculamprotulatientsinensis* (Crosse & Debeaux, 1863)	NC_029210	Wu et al. 2016
MARGARITIFERIDAE		
*Gibbosularochechouarti*i (Heude, 1875)	KX378172	Huang et al. 2018
*Margaritiferafalcata* (Gould, 1850)	NC_015476	Breton et al. 2011
*Cumberlandiamonodonta* (Say, 1829)	NC_034846	Guerra et al. 2017
*Margaritiferadahurica* (Middendorff, 1850)	NC_023942	Yang et al. 2015
HYRIIDAE		
*Echyridellamenziesii* (Dieffenbach, 1843)	NC_034845	Guerra et al. 2017
IRIDINIDAE		
*Muteladubia* (Gmelin, 1791)	NC_034844	Guerra et al. 2017

(*) indicates this species is incertae sedis

### Alignments, partitioning strategies, and phylogenetic analyses

Nucleotide sequences of 12 mitochondrial protein-coding genes (we excluded *atp8*) and 2 rRNA genes were concatenated for construction of the phylogenetic trees. Nucleotide sequences of protein coding genes (PCG) were translated to amino acid sequences using MEGA 5.0 (Tamura et al. 2011), and genes were aligned based on the amino acid sequence (PNGs), or nucleotide sequence (rRNA) using the MUSCLE program (Edgar 2004) with default settings. Alignments of sequences were manually checked and areas of ambiguous alignment were excluded. Finally, 12 PCGs and the 2 rRNA genes were concatenated (11862 bp) using SequenceMatrix (Vaidya et al. 2011). The dataset was then partitioned according to codon position of each PCG and each rRNA gene for phylogenetic analysis. Prior to phylogenetic analysis, a partition homogeneity test was carried out in PAUP* version 4.0b10 (Swofford 2003) to determine rate heterogeneity among genes and codon positions. The partition homogeneity test indicated there was no significant difference in signals (P > 0.05).

PartitionFinder v1.1.1 (Lanfear 2012) was used to select optimal substitution models for the 2 rRNA genes and each codon position of the 12 PCG. Bayesian analyses were undertaken in MrBayes Version 2.01 (Ronquist 2012), four chains were run simultaneously for 1 million generations, and trees were sampled every 1000 generations, with a burn-in of 25%. Stationarity was considered to be reached when the average standard deviation of split frequencies was less than 0.01.

The gene and codon site-based partitioned ML analysis was performed in RAxML implemented in raxmlGUI v.1.3 (Stamatakis 2014), using the GTRGAMMAI model of nucleotide substitution with the search strategy set for rapid bootstrapping. ModelFinder (Chernomor et al. 2016; Kalyaanamoorthy et al. 2017) implemented in IQ-TREE was used to choose the appropriate models, which additionally considers the FreeRate heterogeneity model (+R). IQ-TREE (Nguyen et al. 2015) was also used for ML tree reconstruction, and 1000 ultrafast bootstrap replicates were run to estimate branch support (Minh et al. 2013). The optimal substitution models for each partition by PartitionFinder and ModelFinder are shown in Suppl. material 1: Tables S1, S2.

## Results

### General features of the mitochondrial genomes

**Figure 2.**
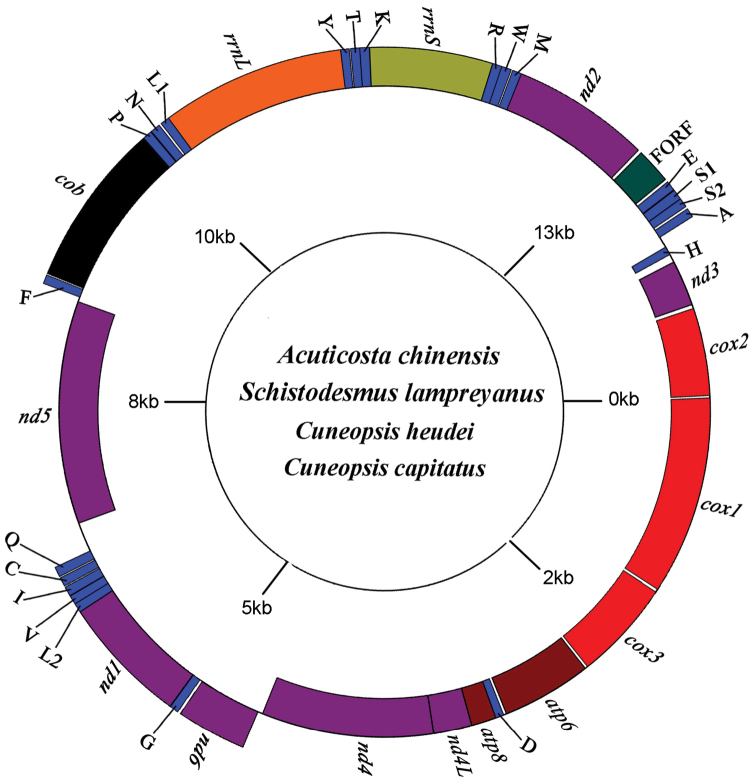
The gene arrangement of the F-type mitochondrial genome of *Acuticostachinensis*, *Schistodesmuslampreyanus*, *Cuneopsisheudei*, and *Cuneopsiscapitatus*.

The lengths of the complete mitogenomes of *Acuticostachinensis*, *Schistodesmuslampreyanus*, *Cuneopsisheudei*, and *Cuneopsiscapitatus* were 15652bp, 15855bp, 15892bp and 15844bp, respectively. The newly sequenced four mitogenomes all contained 13 protein-coding genes, two rRNA genes, 22 tRNAs, and one female specific gene (FORF). All four F-type mitogenomes had the same pattern of gene arrangement. Among the 38 mitochondrial genes, 11 genes were encoded on the heavy chain, and the remaining 27 genes were encoded on the light chain (Figure 2).

The nucleotide composition of the *Acuticostachinensis*, *Schistodesmuslampreyanus*, *Cuneopsisheudei* and *Cuneopsiscapitatus* had obvious A+T bias (*A.chinensis*: 65.73%; *S.lampreyanus*: 64.54%; *C.heudei*: 62.45%; *C.capitatus*: 63.69%). In the base composition analysis for the four species, the A+T skews were negative, and the G+C skew were positive, indicating that the bases composition ratios of the four mitogenomes were T biased to A, and G biased to C. In invertebrate mitochondria, there are three conventional start codons: ATG, ATA and ATT, and three alternative start codons: ATC, TTG, and GTG (Wolstenholme 1992). The mitochondrial genomes of *A.chinensis* and *C.capitatus* had eleven protein coding genes which used the conventional start codons, and the remaining two used alternative start codons. *S.lampreyanus* and *C.heudei* had 12 PCG which used the common start codons, and one used the alternative start codon (Table 4).

**Table 4.** Structural characteristics of F-type mitochondrial genomes of *Acuticostachinensis*, *Schistodesmuslampreyanus*, *Cuneopsisheudei*, and *Cuneopsiscapitatus*. For each protein coding genes, start and stop codons and anticodons are presented in parentheses. Gene lengths are in bp.

	* A. chinensis *	* S. lampreyanus *	* C. heudei *	* C. capitatus *
Total size (bp)	15652	15855	15892	15844
AT%	65.73	64.54	62.45	63.69
CG%	34.27	35.46	37.55	36.31
AT skew	-0.18	-0.19	-0.21	-0.18
GC skew	0.28	0.33	0.33	0.32
No. of NCR	29	27	29	29
No. of overlapping genes	3	3	2	1
Size range of gene overlap	1 to 8	1 to 8	1 to 8	1
*cox1*	1539 (TTG/TAG)	1578 (ATA/TAG)	1566 (TTG/TAA)	1542 (TTG/TAG)
tRNA-Asp (D)	63 (GTC)	64 (GTC)	64 (GTC)	64 (GTC)
*cox3*	780 (ATG/TAA)	780 (ATG/TAA)	780 (ATG/TAA)	780 (ATG/TAG)
*atp6*	702 (ATG/TAA)	702 (ATG/TAG)	702 (ATG/TAG)	702 (ATG/TAG)
*atp8*	189 (ATG/TAA)	192 (ATG/TAA)	192 (ATG/TAG)	192 (ATG/TAG)
*nd4L*	297 (GTG/TAG)	279 (ATG/TAA)	255 (ATG/TAG)	255 (ATG/TAG)
*nd4*	1347 (ATT/TAA)	1347 (ATT/TAA)	1347 (ATT/TAA)	1329 (ATA/TAA)
*nd6*	489 (ATT/TAG)	486 (ATC/TAA)	507 (ATA/TAA)	507 (ATA/TAA)
tRNA-Gly (G)	62 (TCC)	63 (TCC)	63 (TCC))	63 (TCC))
*nd1*	900 (ATA/TAA)	900 (ATA/TAG)	900 (ATA/TAG)	900 (ATA/TAA)
tRNA-Leu (L2)	64 (TAA)	64 (TAA)	63(TAA)	64 (TAA)
tRNA-Val (V)	64 (TAC)	63 (TAC)	63 (TAC)	64 (TAC)
tRNA-Ile (I)	64 (GAT)	67 (GAT)	64 (GAT)	64 (GAT)
tRNA-Cys (C)	64 (GCA)	62 (GCA)	64 (GCA)	61 (GCA)
tRNA-Gln (Q)	69 (TTG)	70 (TTG)	69 (TTG)	69 (TTG)
*nd5*	1728 (ATA/TAA)	1713 (ATA/TAA)	1794 (ATA/TAA)	1734 (ATG/TAA)
tRNA-Phe (F)	66 (GAA)	65 (GAA)	65 (GAA)	64 (GAA)
*Cob*	1137 (ATA/TAA)	1146 (ATT/TAA)	1149 (ATA/TAA)	1020 (ATC/TAA)
tRNA-Pro (P)	64 (TGG)	66 (TGG)	64 (TGG)	64 (TGG)
tRNA-Asn (N)	65 (GTT)	66 (GTT)	68 (GTT)	65 (GTT)
tRNA-Leu (L1)	66 (TAG)	64 (TAG)	63 (TAG)	64 (TAG)
*rrnL*	1285	1304	1302	1297
tRNA-Tyr (Y)	60 (GTA)	61 (GTA)	63 (GTA)	63 (GTA)
tRNA-Thr (T)	61 (TGT)	66 (TGT)	64 (TGT)	63 (TGT)
tRNA-Lys (K)	68 (TTT)	70 (TTT)	70 (TTT)	70 (TTT)
*rrnS*	853	857	859	853
tRNA-Arg (R)	66 (TCG)	67 (TCG)	65 (TCG)	65 (TCG)
tRNA-Trp (W)	65 (TCA)	64(TCA)	63 (TCA)	62 (TCA)
tRNA-Met (M)	65 (CAT)	65 (CAT)	65 (CAT)	65 (CAT)
*nd2*	966 (ATG/TAA)	966 (ATG/TAA)	966 (ATG/TAA)	966 (ATG/TAA)
tRNA-Glu (E)	63 (TTC)	72 (TTC)	68 (TTC)	68 (TTC)
tRNA-Ser (S2)	68 (AGA)	73 (AGA)	68 (TCT)	68 (TCT)
tRNA-Ser (S1)	64 (TGA)	64 (TGA)	64 (CGA)	64 (CGA)
tRNA-Ala (A)	67 (TGC)	65 (TGC)	66 (TGC)	64 (TGC)
tRNA-His (H)	65 (GTG)	69 (GTG)	69 (GTG)	67 (GTG)
*nd3*	357 (ATG/TAG)	357 (ATG/TAG)	357 (ATG/TAA)	357 (ATG/TAG)
*cox2*	681 (ATG/TAA)	681 (ATG/TAG)	681 (ATG/TAA)	681 (ATG/TAG)

The overlapping of neighboring genes is common in freshwater mussel mitochondria. There were three overlaps of neighboring genes in the mitochondrial genome of *Acuticostachinensis* and *Schistodesmuslampreyanus*, and two in *Cuneopsisheudei*. The position of the largest gene overlap (8 bp) was between ND4 and ND4L. The mitochondrial genome of *Cuneopsiscapitatus* only had one overlapping region between tRNA^Met^ and ND2. There were 29 non-coding regions (NCRs) in *A.chinensis*, *C.heudei*, and *C.capitatus*, and 27 NCRs in *S.lampreyanus*. The longest NCRs of the *A.chinensis*, *S.lampreyanus*, *C.heudei*, and *C.capitatus* were 224 bp, 349 bp, 216 bp, and 323 bp, respectively; all were located between ND5 and tRNA^Gln^ (Table 4).

All four mitochondria contained 22 tRNAs, including two serine tRNAs and two leucine tRNAs. The histidine tRNA and aspartate tRNA were located in the heavy chain, whereas the remaining 20 tRNAs were encoded by the light chain. The length of tRNAs differed slightly in each species (Table 4). The tRNA anticodons were the same in all species with the exception of two serine tRNAs. The anticodons of the two serines tRNAs of *A.chinensis* and *S.lampreyanus* were AGA and TGA, while those of *C.heudei* and *C.capitatus* were TCT and CGA (Table 4).

### Phylogenetic analyses

**Figure 3.**
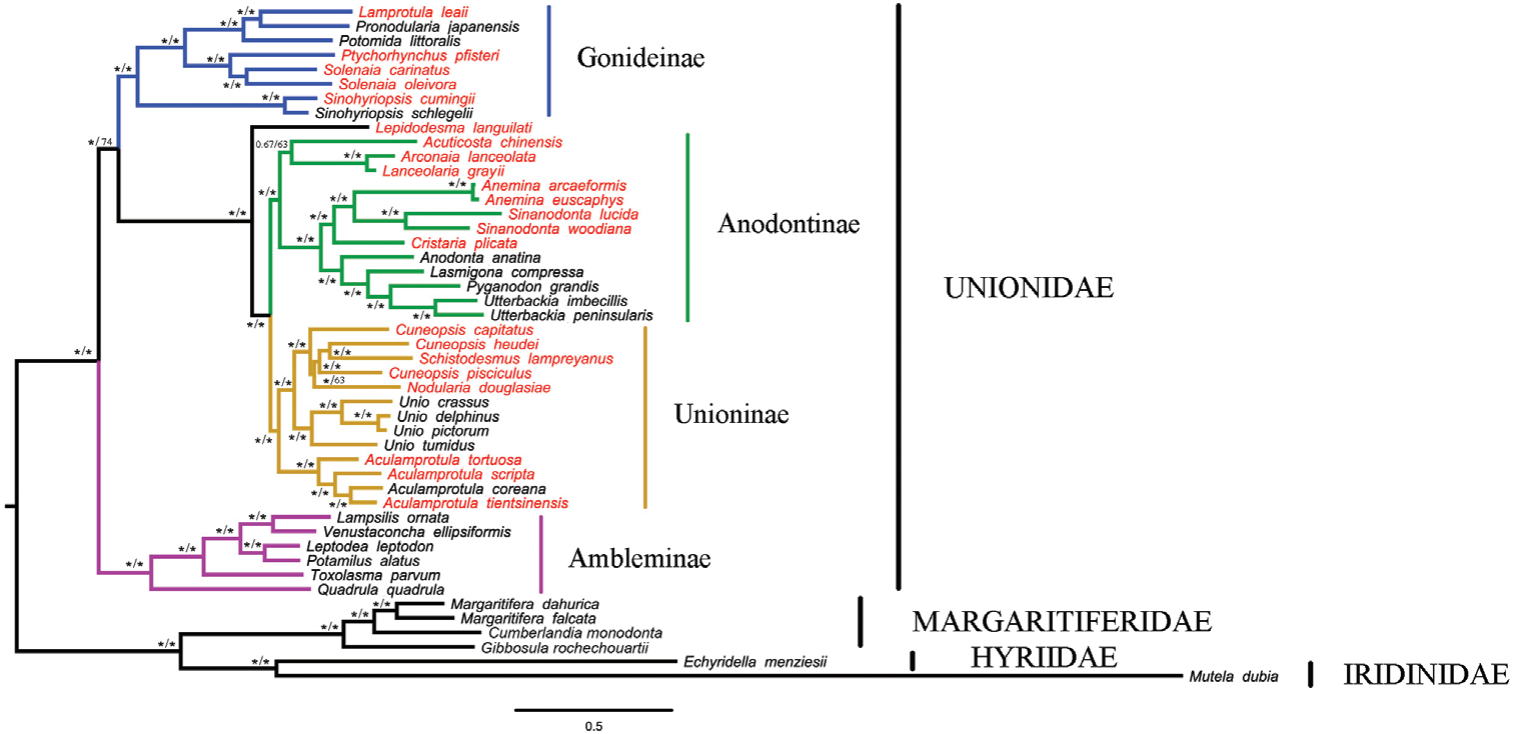
Phylogenetic trees of freshwater mussels obtained by Bayesian Inference (BI) and Maximum Likelihood (ML) analyses of 12 mitochondrial protein-coding gene sequences (except *atp8*) and two rRNA combined dataset. Support values above the branches are posterior probabilities and bootstrap support. (*) indicates 100 percent bootstrap support and posterior probabilities. Red font indicates Chinese species.

ML and BI trees have completely congruent topologies and in general are well supported by high bootstrap and posterior probability values at almost all nodes (Figure 3). The mitogenomic dataset supports the monophyly of four Unionidae subfamilies (i.e., Unioninae, Anodontinae, Ambleminae, and Gonideinae) by both ML and BI methods. Phylogenetic analyses reveal the following relationships: (((Unioninae + Anodontinae) + Gonideinae) + Ambleminae) within the Unionidae.

Our phylogenetic analyses indicate that except for *Lepidodesmalanguilati* (Heude, 1874), the 21 Chinese species belong to the following three subfamilies: Unioninae (*Aculamprotula*, *Cuneopsis*, *Nodularia* and *Schistodesmus*), Anodontinae (*Cristaria*, *Arconaia*, *Acuticosta*, *Lanceolaria*, *Anemina* and *Sinoanodonta*), and Gonideinae (*Ptychorhynchus*, *Solenaia*, *Lamprotula*, *Sinohyriopsis*). Our results support the placement of *Acuticostachinensis* in the Anodontinae, but *Leidodesmalanguilati* is not placed as a member of any subfamily, but instead is the well-supported sister taxon to the monophyletic group formed by the Unioninae and Anodontinae.

## Discussion

### Phylogenetic relationships of subfamilies in the Unionidae

**Figure 4.**
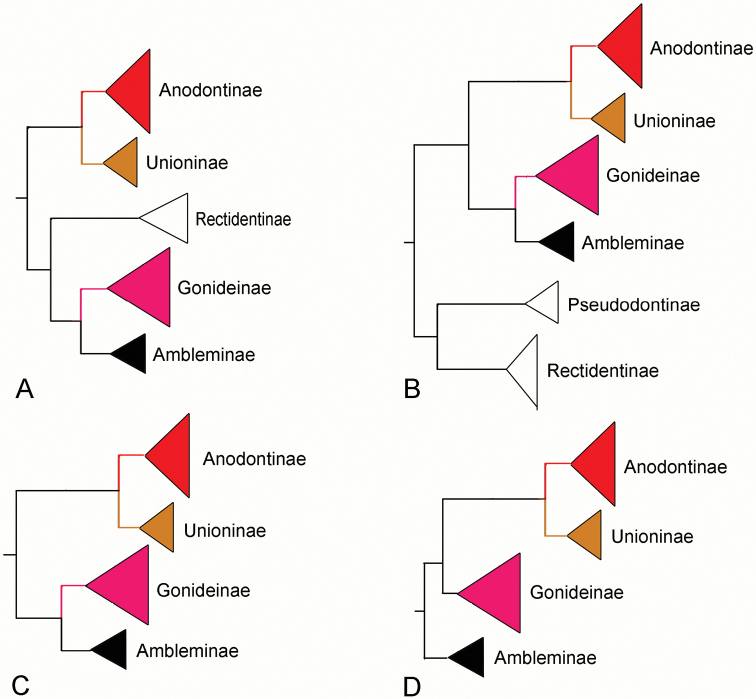
Hypotheses of phylogenetic relationships among subfamilies of the Unionidae form this and other studies. **A**Lopes-Lima et al. (2017a)**B**Bolotov et al. (2017a)**C**Huang et al. 2013; Burzyński et al. 2017; Huang et al. 2018; Wu et al. 2016, 2017b**D** This study.

In this study, we provide a novel phylogenetic hypothesis for relationships between subfamilies in the Unionidae (Figure 4). Other phylogenetic analyses of the Unionidae have been based on selected gene regions. For example, Lopes-Lima et al. (2017a) proposed the phylogenetic relationship of the subfamily based on COI and 28S as follows: (Anodontinae + Unioninae) + (Rectidentinae + (Ambleminae + Gonideninae)). Bolotov et al. (2017a) proposed relationships based on three loci (COI, 16S and 28S), and adding more taxa: ((Anodontinae + Unioninae) + (Ambleminae + Gonideninae)) + (Rectidentinae + Pseudodontinae). Prior investigations into subfamily relationships in the Unionidae, based on complete mitochondrial genomes, seem to be consistent with these earlier studies, (Anodontinae + Unioninae) + (Ambleminae + Gonideninae) (Huang et al. 2013; Burzyński et al. 2017; Huang et al. 2018; Wu et al. 2016, 2017b). The current study is based on the mitochondrial genome sequences for the largest number of unionid species (41). By increasing the number of taxa and the amount of DNA sequences, we obtain a unique set of phylogenetic relationships: ((Anodontinae + Unioninae) + Gonideninae) + Ambleminae). Our phylogeny differs from other studies based on mitochondrial genome sequences in that the Ambleminae is the basal subfamily as opposed to the sister Gonideninae.

Bolotov et al. (2017a) proposed that the most recent common ancestor (MRCA) of the Anodontinae, Unioninae, Ambleminae, and Gonideninae likely originated in East Asia (Probability 65.8%). Under this scenario the MRCA of Anodontinae + Unioninae arose in East Asia during the Cretaceous period, whereas the MRCA of Ambleminae + Gonideninae was continuously distributed in East Asia and North America. The ancestor of the Ambleminae was most likely to originate in North America. The diversification of each subfamily occurred in the late Cretaceous (Bolotov et al. 2017a). The results of phylogenetic analyses in the current study have different evolutionary implications. Our results indicate that the Ambleminae is basal to the other three subfamilies, and its origin is therefore earlier than the other three subfamilies. Globally, eight subfamilies (Anodontinae, Unioninae, Pseudodontinae, Gonideinae, Ambleminae, Rectidentinae, Parreysiinae, and Modellnaiinae) are recognized in the Unionidae (Bolotov et al. 2017a; Lopes-Lima et al. 2017a; Whelan et al. 2011). The lack of mitochondrial genomes for Rectidentinae, Parreysiinae, Modellnaiinae, and Pseudodontinae, precluded their incorporation into this study. However, we believe that the fully resolved phylogenetic tree, with high branch support in the present study, serves as a framework for further studies on the Unionidae, Future phylogenetic analyses based on complete mitochondrial genome sequences of representatives of all the subfamilies in the Unionidae will ultimately produce well-supported phylogenetic hypotheses for the Unionidae.

### Phylogeny and taxonomy of Chinese taxa

The classification of the Chinese unionid genera has been in a state of flux, different studies having placed the same genus in different subfamilies. For example, based on the presence or absence of the glochidial hooks and the type of marsupium, Wu et al. (1999a) divided the genus *Lamproulasensu lato* Simpson, 1900 into *Lamprotulasensu stricto* and *Aculamprotula* Wu, Liang, Wang & Ouyang, 1999. This distinction was later confirmed by molecular data (Zhou et al. 2007; Pfeiffer and Graf 2013; Wu et al. 2018b), but the classification of *Lamprotula* has also been disputed. Our results do not support the taxonomy of Huang et al. (2002), Zhou et al. (2007) and Ouyang et al. (2011; 2015) that placed *Lamprotula* sensu stricto in the Ambleminae. Our phylogenetic analyses instead confirm the results of Pfeiffer and Graf (2013), Lopes-Lima et al. (2017a), Bolotov et al. (2017a; b) and Wu et al. (2018b) that *Lamprotula* is a member of the Gonideninae. The classification of the genus *Sinohyriopsis* has also been unstable. The shape of the glochidia of *Sinohyriopsiscumingii* (Lea, 1852) is semi-elliptical and unhooked, and resembles the typical morphology of glochidia in the Gonideninae (Wu et al. 2018a). But the marsupium of *S.cumingii* is restricted to the outer two demibranchs of the gills (ectobranchous), whereas in other species in the Gonideninae (*Lamprotulaleaii* (Griffith & Pidgeon, 1833) *Solenaiacarinatus* (Heude, 1877) and *Solenaiaoleivora* (Heude, 1877)) the marsupium includes all four demibranchs (tetragenous) (Wu et al. 2018a). Therefore, based on anatomical features alone, the classification of the *Sinohyriopsis* in the Gonideninae has always been in doubt. Prior phylogenetic analyses based on one or two mitochondrial molecular markers (Huang et al. 2002; Zhou et al. 2007; Ouyang et al. 2011; 2015) placed *Sinohyriopsis* in the Ambleminae, However, our results indicate that *Sinohyriopsis* should be placed in the Gonideninae, confirming the conclusions of Lopes-Lima et al. (2017a) and Bolotov et al. (2017a, b). The placement of *Aculamprotula* has not been as controversial and our results place it in the Unioninae.

The genus *Lepidodesma* Simpson, 1896 is endemic to China and *Lepidodesmalanguilati* (Heude, 1874) is the type species. The juvenile of this species is thin and fragile, and the adult shell is robust. In addition, adults lack pseudocardinal teeth, but possess lateral teeth and the glochidia are triangular and have hooks. The breeding period is from February to August, and the type of marsupium is ectobranchous (Wu et al. 2018a). These characteristics are similar to species in the subfamily Unioninae and Anodontinae. Other characters, such as the size of the glochidia, which is large, and the tripartite water tubes (Wu et al. 2018a), indicate an affinity with the subfamily Anodontinae. The classification of *Lepidodesma* has alternated between these two subfamilies with some (Simpson 1900, Huang et al. 2002, Graf and Cummings 2007, Zhou et al. 2016) placing it in the Unioninae, and others (Haas 1969, Liu et al. 1979, Prozorova et al. 2005) in the Anodontinae. The results of our study indicate a novel result in which *L.languilati* is place in neither of these subfamilies, but is sister to a clade that includes both the Unioninae and Anodontinae. The robust branch support values indicate that *L.languilati* is not a member of either subfamily, but is instead a member of another, as yet unrecognized clade or perhaps is the remnant of a once larger more diverse group. Owing to the lack of available mitochondrial genomes for representatives of the Rectidentinae, Parreysiinae, and Pseudodontinae, our study did not include these subfamilies, and we recognize that their inclusion could produce a different set of relationships.

Due to the emphasis on the morphological characteristics of the shell, malacologists have consistently supported including both *Arconaia* and *Lanceolaria* in the Unioninae (Haas 1969; Liu 1979; Graf and Cummings 2007). The shells of *Arconaia* and *Lanceolaria* are thick and have distinct hinge teeth, and the morphology of the glochidia (triangular; hooked) and type of marsupium (ectobranchous) are similar to species of the subfamily Unioninae and Anodontinae (Wu et al. 2018a). The phylogenetic relationships inferred by different molecular markers, seem to confirm the phylogenetic position of these genera in the Unioninae (Huang et al. 2002; Zhou et al. 2007; Ouyang et al. 2015). However, the above-mentioned phylogenetic analyses included a limited number of taxa, and several key nodes in the phylogeny had low branch support. The results of the current study support the placement of *Arconaia* and *Lanceolaria* in the Anodontinae, confirming the results of Lopes-Lima et al. (2017a) and Wu et al. (2018b).

The genus *Acuticosta* was erected by Simpson and *Acuticostachinensis* (Lea, 1868) was designated as the type species. Based on the marsupium, anatomy, larvae type and umbo sculpture, Simpson (1900) placed this genus in the Hyriinae. Subsequently, Chinese malacologists (Liu et al. 1979) re-classified the genus as a member of the Unioninae based on the presence or absence of hinge teeth. Prozorova et al. (2005) in a review of the bivalves in the Yangtze River drainage, placed the genus in Acuticostinae, although Graf and Cummings (2007) still maintained *Acuticosta* in the Unioninae. Molecular genetic analyses of a variety of markers by Huang et al. (2002), Zhou et al. (2007), and Ouyang et al. (2011; 2015) all indicated that *A.chinensis* was a member of the Unioninae. However, the limited taxon sampling and low branch support values in molecular phylogenetic analyses have allowed questions concerning the true affinities of *Acuticosta* to persist (Pfeiffer and Graf 2013; Huang et al. 2013; Lopes-Lima et al. 2017). Recently, Wu et al. (2018b) indicated that *A.chinensis* is a member of the Anodontinae based on mitochondrial DNA sequences of two genes. The current analysis of mitochondrial genomes provides further support for the placement of *Acuticosta* in the Anodontinae and indicates affinity of *Acuticosta* to the genera *Arconaia* and *Lanceolaria*.

### Endangered status and conservation implications

China is a vast territory with a huge number of lakes and rivers. As a result, it is one of the most species-rich regions in the world (Zieritiz et al. 2017; Cai et al. 2018). However, in recent decades, freshwater mussels in China have declined drastically, and species diversity has been seriously threatened. At present, 40 species of Chinese unionids are included in the 2018 IUCN Red List, although 32 of these are categorized as data deficient or least concern. In addition, nearly half of the species included had not been evaluated. At present, advancing urbanization in the Yangtze River Basin, increasingly threatens the habitat of freshwater mussels, and conservation and management efforts targeting freshwater taxa are urgently needed.

Understanding of the phylogenetic diversity of freshwater mussels has important significance for determining the priority conservation strategies of species (Lopes-Lima et al. 2017b, 2018). This study provides support for the classification of a number of Chinese species, and lays the foundation for the future development of a more comprehensive phylogenetic based classification for freshwater unionids in China. Accurate taxonomic placement of rare and understudied species is central to many aspects of conservation as important biological characteristics (e.g., habitat preferences, reproductive traits) can be inferred from closely related taxa. Future research on Chinese unionids should focus on species delimitation and classification. In addition, more research is needed on understanding the basic ecology of Chinese mussels including species distributions, habitat preferences, and host fish identification.

## Supplementary materials

Supplementary material 1 Supplementary Tables S1, S2 Data type: molecular data Explanation note: Table S1: Partitioning strategies from PartitionFinder for mt genome dataset; Table S2: Partitioning strategies from ModelFinder for mt genome dataset. This is an open access article distributed under the terms of the Creative Commons Attribution License (CC BY 4.0), which permits unrestricted use, distribution, and reproduction in any medium, provided the original author and source are credited.Rui-Wen Wu, Xiong-Jun Liu, Sa Wang, Kevin J. Roe, Shan Ouyang, Xiao-Ping Wu
