# Supplementary material for: Analysis of mitochondrial genomes resolves the phylogenetic position of Chinese freshwater mussels (Bivalvia, Unionidae)
Source: Zookeys. 2019 Jan 3;(812):23–46. doi: 10.3897/zookeys.812.29908 (PMC6328525; doi:10.3897/zookeys.812.29908)
Supplement: Supplementary material 1 [file zookeys-812-023-s001.docx]

Supplementary Table S1. Partitioning strategies from PartitionFinder for mt genome dataset.

| Subset | Best Model | Partition names |
| --- | --- | --- |
| 1 | GTR+I+G | 12S, 16S |
| 2 | GTR+I+G | ND4_1, ND5_1, ND3_ 1, ATP6_1, ND4L_1, ND4L_2 |
| 3 | GTR+I+G | ND5_2, ND4_2, ATP6_2 |
| 4 | GTR+I+ G | ND5_3, ATP6_3, ND4_3 |
| 5 | GTR+I+G | CO1_1, CO2_1, CO3_1 |
| 6 | GTR+I+G | CO1_2, CO2_2, CO3_2 |
| 7 | GTR+I+G | CO2_3, CO1_3, CO3_3, ND4L_3, ND3_3 |
| 8 | SYM+I+G | CYTB_1, ND1_1 |
| 9 | GTR+I+G | CYTB_2, ND1_2 |
| 10 | GTR+G | CYTB_3, ND1_3, ND6_3 |
| 11 | GTR+I+G | ND6_1, ND2_1 |
| 12 | GTR+I+G | ND6_2, ND2_2 |
| 13 | HKY+I+G | ND2_3 |
| 14 | GTR+I+G | ND3_1, ND3_2 |

Supplementary Table S2. Partitioning strategies from ModelFinder for mt genome

dataset.

| Subset | Best Model | Partition names |
| --- | --- | --- |
| 1 | TIM2+F+I+G4 | 12S rRNA |
| 2 | TIM2+F+I+G4 | 16 S rRNA |
| 3 | HKY+F+I+G4 | ATP6_1 |
| 4 | TVM+F+G4 | ATP6_2 |
| 5 | TPM3u+F+G4 | ATP6_3 |
| 6 | TN+F+I+G4 | CO1_1 |
| 7 | TVM+F+I+G4 | CO1_2 |
| 8 | TPM2u+F+G4 | CO1_3 |
| 9 | HKY+F+G4 | CO2_1 |
| 10 | TVM+F+I+G4 | CO2_2 |
| 11 | TPM2u+F+I+G4 | CO2_3 |
| 12 | K3Pu+F+I+G4 | CO3_1 |
| 13 | TPM2u+F+I+G4 | CO3_2 |
| 14 | TPM2u+F+I+G4 | CO3_3 |
| 15 | TIM2e+I+G4 | CYTB_1 |
| 16 | TPM3u+F+I+G4 | CYTB_2 |
| 17 | TIM3+F+ASC+G4 | CYTB_3 |
| 18 | TIM2e+I+G4 | ND1_1 |
| 19 | TVM+F+I+G4 | ND1_2 |
| 20 | HKY+F+ASC+G4 | ND1_3 |
| 21 | TN+F+I+G4 | ND2_1 |
| 22 | GTR+F+I+G4 | ND2_2 |
| 23 | HKY+F+I+G4 | ND2_3 |
| 24 | TIM2+F+G4 | ND3_1 |
| 25 | HKY+F+I+G4 | ND3_2 |
| 26 | TIM3+F+ASC+G4 | ND3_3 |
| 27 | TPM2u+F+I+G4 | ND4_1 |
| 28 | TVM+F+I+G4 | ND4_2 |
| 29 | TIM+F+G4 | ND4_3 |
| 30 | TN+F+G4 | ND4L_1 |
| 31 | K3Pu+F+G4 | ND4L_2 |
| 32 | HKY+F+I+G4 | ND4L_3 |
| 33 | TIM2+F+I+G4 | ND5_1 |
| 34 | GTR+F+I+G4 | ND5_2 |
| 35 | TIM3+F+G4 | ND5_3 |
| 36 | HKY+F+I+G4 | ND6_1 |
| 37 | TIM3+F+I+G4 | ND6_2 |
| 38 | TN+F+ASC+G4 | ND6_3 |
